# Management Strategies and Outcomes for VHL-related Craniospinal Hemangioblastomas

**DOI:** 10.15586/jkcvhl.2017.90

**Published:** 2017-08-28

**Authors:** Christ Ordookhanian, Paul E. Kaloostian, Samer S. Ghostine, Philippe E. Spiess, Arnold B. Etame

**Affiliations:** 1 Department of Neurological Surgery, University of California at Riverside School of Medicine, Riverside, CA, USA; 2 Department of Urologic Oncology, Lee Moffitt Cancer Center and Research Institute, Tampa, FL, USA; 3 Department of Neuro-Oncology, H. Lee Moffitt Cancer Center and Research Institute, Tampa, FL, USA

**Keywords:** craniospinal hemangioblastoma, natural history, radiographic diagnosis, surgical resection, von Hippel–Lindau syndrome

## Abstract

Hemangioblastomas are rare and benign tumors accounting for less than 2% of all central nervous system (CNS) tumors. The vast majority of hemangioblastomas occur sporadically, whereas a small number of cases, especially in younger patients, are associated with Von Hippel–Lindau (VHL) syndrome. It is thought that loss of tumor suppressor function of the VHL gene results in stabilization of hypoxia-inducible factor alpha with downstream activation of cellular proliferative and angiogenic genes that promote tumorigenesis. VHL-related hemangioblastomas predominantly occur in the cerebellum and spine. Lesions are often diagnosed on contrast-enhanced craniospinal MRIs, and the diagnosis of VHL occurs through assessment for germline VHL mutations. Surgical resection remains the primary treatment modality for symptomatic or worrisome lesions, with excellent local control rates and neurological outcomes. Stereotactic radiotherapy can be employed in patients who are deemed high risk for surgery, have multiple lesions, or have non-resectable lesions. Given the tendency for development of either new or multiple lesions, close radiographic surveillance is often recommended for asymptomatic lesions.

## Introduction

Hemangioblastomas (HB) are rare low-grade vascular tumors within the central nervous system (CNS) identified by the World Health Organization (WHO) criteria as Grade I tumors and account for 1–2% of CNS tumors (1). They consist of closely packed capillaries within proliferative stromal cells which form the main neoplastic component and harbor the genetic defect (2). HB can occur as solitary sporadic tumors or as multiple familial tumors associated with von Hippel–Lindau (VHL), an autosomal dominant disorder (3). Some of the key clinical and genetic epidemiologic features of VHL syndrome are highlighted in Table 1 (4). The overwhelming majority of HB occur sporadically (3). VHL-related HB arise from defects associated with loss of tumor suppression function of the VHL gene (3, 5–7). Multiple HB can be seen in at least 60% of VHL patients (8–12). The most common locations for VHL-HB within the CNS include the posterior fossa and spinal cord (1). Given the locational CNS predilection, HB can be a significant source of morbidity and mortality in VHL patients. In fact, the most common cause of demise in VHL patients appears to be from posterior fossa hemorrhages associated with HB and also renal cell carcinoma (RCC), which is also part of the syndrome (8, 13, 14). Surgical resection of symptomatic or radiographically progressing lesions can be curative.

**Table 1:** Epidemiology-related parameters of VHL

VHL degree of incidence	1 in 36,000
VHL point prevalence	1 in 38,000
Age range of diagnosis (years)	Infancy to 70
Average age of diagnosis (years)	26–29
Average age for full penetrance of VHL	70
Male:female penetrance	1:1
*De novo* VHL mutations	20%
Familial VHL mutations	80%
Common clinical manifestation	Presenting in VHL cases (%)
CNS HB	30–80
Renal cell carcinoma	30–70
Renal cysts	60
Retinal angiomas	15–70
Endolymphatic sac tumors	3–16
Pancreatic cyst	20–70
Pancreatic neuroendocrine tumor	15–56
Pheochromocytoma	16

## Pathogenesis of HB in VHL

VHL is an autosomal dominant syndrome with age-related penetrance, characterized with over 300 germ-line mutations of the *VHL* gene on the short arm of chromosome 3p25 (15, 16). Deletion of a single copy of the VHL gene, also known as loss of heterozygosity, can predispose patients to the syndrome. Approximately one-third of VHL cases involve deletion mutations, whereas the remaining two-thirds are related non-deletion mutations (15, 16). The subsequent loss of the tumor suppressive function of VHL secondary to inactivation of both alleles has been postulated as the basis for the development of neoplasms such as HB, pheochromocytomas, and RCC in patients with VHL (17, 18). Although the vast majority of VHL cases have a familial genetic pattern, the syndrome can also manifest de novo without a prior family history. In a large series of 181 patients with VHL evaluated at the National Institutes of Health (NIH), 41 patients did not have a family history diagnosis of VHL suggesting a de novo manifestation (19). However, although VHL germline mutations might not be apparent when assessed by standard techniques such as Southern blot and gene sequencing, further analysis of segments of peripheral blood lymphocytes using additional molecular techniques might uncover the mutation, hence underscoring the significance of genetic mosaicism (19). It has, therefore, been postulated that parental mosaicism might account for some of the de novo or sporadic cases of VHL (19).

The main function of the VHL gene product (pVHL) is regulation through ubiquitination of hypoxia-inducible factor alpha (HIF-α), the major mediator of the cellular response to hypoxia (20–22). In the presence of oxygen, the proline residues of HIF-α are hydroxylated by prolyl hydroxylase enzymes (20, 21, 23). The hydroxylation provides a ubiquitination tag on HIF-α for the VHL complex. VHL protein forms a VHL complex through interaction of its binding domains with elongation factors (elongin C and elongin B), and cullin-2 (20–22). Binding of the beta domain of this VHL complex with prolyl-hydroxylated HIF-α results in ubiquitination and degradation of HIF-α (24). However, prolyl-hydroxylation of HIF-α does not occur in hypoxic conditions (25, 26). As such, the VHL protein complex does not recognize and bind to HIF-α. Similarly, defects in VHL protein complex prevent its regulation and degradation of HIF-α. Hence, it is postulated that aberrancies in VHL leading to stabilization of HIF-α result in downstream up-regulation of hypoxia response genes, such as vascular endothelial growth factor (VEGF) and platelet-derived growth factor (PDGF), that have a role in neoplastic transformation (25, 27, 28).

Although genetic defects in VHL can predispose to HB in the craniospinal axis, the genetic mechanisms through which HB develop are not fully elucidated. Besides VHL mutations, germline allelic variations of several genes including CCND1, MMP1, and MMP3 have been implicated in pathogenesis of HB (29). Several studies that examined tissue expression of EGFR in HB demonstrated universally that there was an over-expression of EGFR in HB (30–32). This is not surprising because EGFR plays a role in cell proliferation and angiogenesis. A more recent large study of 44 HB samples using droplet digital polymerase chain reaction and high-resolution single nucleotide polymorphism (SNR) arrays implicated 23 candidate in the pathogenesis of HB (33). The candidate genes included the following: EGFR, PRDM16, PTPN11, HOXD11, HOXD13, FLT3, PTCH, FGFR1, FOXP1, GPC3, HOXC13, HOXC11, MKL1, CHEK2, IRF4, GPHN, IKZF1, RB1, and HOXA9, and micro RNA, such as hsa-mir-196a-2. The most common aberrations were deletion of CHEK2 and amplifications of EGFR, PTPN11, and PTCH. In general, the genes implicated are functionally involved with cell proliferation and angiogenesis, which accounts for the highly vascular phenotype of HB.

## Clinical presentation

Patients with HB can be asymptomatic or symptomatic based on the location and mass effects of the tumor (3, 34, 35). Lesions in the posterior fossa can hemorrhage and cause obstructive hydrocephalus and associated symptoms of nausea, headaches, ataxia and profound lethargy. In extreme situations, brainstem compression with subsequent herniation has been reported (36). Moreover, even small hemorrhages in brainstem lesions can result in profound neurological symptoms. In general, symptomatic lesions were usually associated with a large cyst and brain edema (37).

Spinal HB can be asymptomatic, associated with pain, or produce myelopathic symptoms of gait impairment, paralysis, sensory deficits, bowel, and bladder dysfunction. Pain often precedes myelopathy (38, 39). Myelopathic symptoms are usually related to compression of long tracts with the spinal cord from tumor hemorrhage. Less commonly, patients can present with spinal subarachnoid hemorrhage (39, 40). Leptomeningeal dissemination of HB is an extremely rare presentation that has been reported in a handful of cases (41–45).

## Radiographic diagnosis of HB

**Figure 1:**
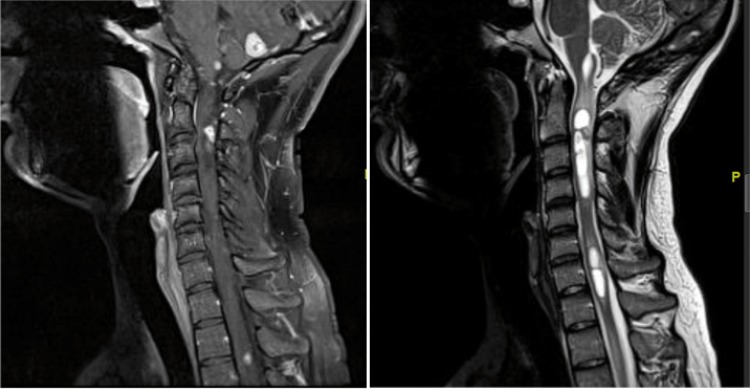
Sagittal MRI of posterior fossa and cervical spine. *Left*: T1 with gadolinium shows multiple enhancing HB tumors involving cerebellum, brainstem, and spinal cord. *Right*: T2 MRI shows multiple intramedullary cystic lesions consistent with VHL hemangioblastoma.

HB represents one of the earliest manifestations of VHL syndrome (11). Gadolinium-enhanced magnetic resonance imaging (MRI) is the best diagnostic modality for HB because its resolution is markedly superior to CT (46, 47). They appear as homogenously enhancing lesions and are sometimes associated with a cystic component. Hemorrhage might be present. Tumors can be seen in the brainstem, cerebellum, or spinal cord (Figure 1). Spinal cord lesions can exist on the pial surface as small enhancing nodules or intramedullary with associated syrinx. Symptomatic spinal HB are often associated with syrinx and edema (48). Larger spinal HB are more likely associated with flow voids compared to smaller HB (48). Patients who are known to be at risk for VHL can be screened with MRI of the neural axis. Similarly, if a lesion is noted on brain MRI, then an MRI of the spine is recommended.

Alternative diagnostic modalities include contrast-enhanced CT scans and angiography (49–51). CT might be employed where there are contraindications to MRI. Homogenously enhancing nodules can be seen on the pial surface with CT. CT can also assess the extent of cystic compression, hemorrhage, and hydrocephalus in the case of large posterior fossa lesions. Angiography can demonstrate the classic tumor vascular blush.

## Natural history of HB

Several studies have examined the natural history of HB as it relates to the development of symptoms and optimal timing of interventions (1, 35, 52). Because some HB lesions might remain asymptomatic for very long periods of time, it is important to avoid unnecessary surgeries in this patient population.

Wanebo et al. retrospectively examined 160 consecutive patients with VHL through correlating clinical history with serial volumetric analysis of tumors on MRI (52). They noted a predilection of cystic lesion towards the cerebellum, spinal cord, and brainstem. Compared to the associated tumor nodule, the cysts expanded faster and were the basis for neurological symptoms as opposed to the actual tumor. What was also noteworthy was the fact that a significant number of untreated asymptomatic lesions maintained their status quo for many years. From the above study, it appears that cystic lesions should be cautiously observed and considered for treatment whenever there is substantial interval expansion with risks to vital structures.

In another retrospective study of 158 patients with VHL over a 10-year span, Ammerman and colleagues reported symptomatic lesions in only 41% of patients over that time span (1). The vast majority of lesions remained asymptomatic even with radiographic progression. The authors used clinical symptoms as opposed to radiographic progression as the basis for surgical intervention. They concluded that such an approach spared each patient from four additional unnecessary procedures over that 10 years time span.

The natural history of HB has been assessed in a prospective format as well. Lonser and colleagues prospectively enrolled 225 VHL patients with HB to assess for factors associated with tumorigenesis and neurological symptoms (53). Their assessment of increased tumors burden revealed a predilection for male sex and partial deletions in VHL gene. In addition, younger patients were most likely to develop new tumors. Furthermore, rapidly growing tumors were most likely encountered in male patients, most likely symptomatic, and associated with a cyst. Hence, the above considerations should be taken into account in the management of treatment of VHL patients with HB.

## Surgical resection of craniospinal HB

Surgery is the preferred treatment modality of HB given the low morbidity of surgery. Surgery can relieve compressive neurological symptoms and can be curative. Although the role of surgery is clear for symptomatic or large lesions, surgery for asymptomatic lesions is debatable (52, 53). Some have advocated for treating intramedullary lesions at the onset of diagnosis as opposed to the development of neurological symptoms based on the observation that patients are least likely to improve after surgery following neurological deterioration (53–55). However, a recent prospective study on the natural history of 1921 CNS HB in 225 VHL patients found that the vast majority of asymptomatic lesions progressed in a stepwise fashion whereby neither absolute tumor size nor rate of tumor growth was the universal determinant of neurological symptoms (35). The authors concluded that surgery for HB should only be recommended at the onset of neurological symptoms (35).

Surgical resection of HB is similar to resection of any vascular malformation. Because HB are highly vascular lesions, the goal of resection entails circumferential dissection along the interface between tumor and brain without prematurely violating the tumor. Arterial feeders are circumferentially coagulated and disconnected resulting in an en-bloc resection with risks for intraoperative as well as postoperative hemorrhage. A midline myelotomy offers the safest approach that minimizes damage to the posterior columns. In some instances, preoperative embolization could be considered in order to minimize postoperative blood loss (56–58). Because spinal lesions are generally intramedullary, neurophysiologic monitoring of sensory and motor evoke potentials should be incorporated as part of the resection strategy. Similarly, lesions in the brainstem might warrant monitoring of the above modalities as well as brainstem monitoring.

As previously mentioned, surgical resection of HB can be curative with less morbidity at experienced centers. There are tumor-related characteristics that can significantly impact outcomes. In one study, solid tumor configuration, but not tumor size, was noted to be the key determinant of immediate or long-term postoperative outcomes in patients with cerebellar HB (59). For instance, in that study patients with solid tumors had a propensity for postoperative hematomas requiring surgical intervention. In terms of long-term outcomes, patients with solid tumors had a markedly negative outcome compared to those with solid tumors. Because solid tumors are very vascular, preoperative embolization could minimize the chances of postoperative hematomas in the posterior fossa.

Favorable outcomes have been reported for tumors in the medulla and spinal cord when the onset of symptoms was the main indication for surgical intervention. Parker and colleagues (60) reported their experience in 34 patients with HB and a mean age of 41. They attained gross total tumor resection in approximately 85% of cases. There were no mortalities from surgery, and less than 18% of patients experienced worsening of symptoms following surgery. In another study of 14 patients with 15 brainstem HB lesions, Pavesi and colleagues (61) reported that although patients with brainstem lesions were more likely to have immediate postoperative deficits, the long-term outcomes were highly favorable. For instance, following surgery, 9 out of 14 patients experienced transient neurological deficits. However, at long-term follow-up, at least 10 patients had performance levels superior to their preoperative performance. The role of intraoperative neurophysiological monitoring (IONM) in improving outcomes during resection of spinal HB has been evaluated and emphasized (62). In a series of 24 patients who underwent surgeries for 27 lesions, the authors noted a strong correlation between a pathological IONM findings and an adverse outcome (62). On the contrary, patients who had nonpathological IONM findings were significantly less likely to have new sensorimotor deficits following surgical resection.

## Radiotherapy for HB

In situations where patients are not good surgical resection candidates or where lesions are not amenable to safe resection, stereotactic radiation is a favorable option. Radiation can also be used to address multiple lesions as well as in the setting of tumor recurrence. Although not a curative strategy, it can provide reasonable and sustained local tumor control (63–68).

Kano and colleagues published one of the largest series evaluating the long-term outcomes of stereotactic radiosurgery (SRS) in the management of HB consisting of 186 patients with 517 lesions (64). They reported overall survival rates of 94% at 3 years, 90% at 5 years, and 74% at 10 years. The associated tumor control rates were 92% at 3 years, 89% at 5 years, and 79% at 10 years. Hence, excellent local control rates are feasible with SRS.

However, it is worthwhile noting that there appears to be differential response to intracranial SRS for sporadic versus VHL-related HB. In their assessment of long-term outcomes in 57 intracranial HB treated with SRS, Hanakita and colleagues reported 5- and 10-year tumor control rates of 67 and 44%, respectively, for sporadic HB compared to 97 and 83% , respectively, for VHL-related HB (67). Besides VHL pathology, SRS was much effective for small and solid tumors compared to large and cystic tumors.

SRS is equally effective for spinal HB in terms of halting tumor progression and improving neurological symptoms associated with HB. Pan and colleagues assessed radiographic and clinical outcomes in 34 spinal HB tumors (66). Following SRS treatment, 94% of the tumors were either stable or regressed with local control rates at 1, 3, and 5 years being 96, 92, and 92%, respectively. Symptom improvement was associated with 81% of treated lesions. Hence, SRS was deemed as a safe approach for spinal HB.

A recent systematic review comparison of retrospective data of surgical resection versus SRS for spinal HB showed that only 2% of tumors treated with SRS actually progressed (69). SRS was associated with minimal side effects. The same study showed that surgical resection resulted in successful removal of tumor with a recurrence rate of approximately 5%. It was also evident that at least 96% of patients were either clinically stable or improved on long-term follow-up from surgery. It is worthwhile noting that no statistical comparisons could be done between the surgery and SRS cohort which is a major limitation.

Overall, SRS for HB appears to be an effective alternative strategy whenever safe surgical resection is not practical. It appears to be more effective for VHL patients with HB, small tumors, and solid lesions.

## Conclusion

Although HB are benign tumors, they can cause significant neurological impairment or even mortality following intratumoral hemorrhage or cystic expansion of tumor. Because the vast majority of lesions are asymptomatic with very minimal growth, observation is reasonable. Factors associated with tumor progression and treatment outcomes should be considered in the timing of interventions. Lesions that are symptomatic or demonstrate worrisome radiographic features warrant surgery resection if safely feasible. Radiosurgery remains an acceptable alternative to surgical resection. Excellent long-term outcomes can be expected with surgery and radiation.

## Conflict of interest

The authors declare no potential conflicts of interest with respect to research, authorship and/or publication of this article.
